# MLST Subtypes and Population Genetic Structure of *Cryptosporidium andersoni* from Dairy Cattle and Beef Cattle in Northeastern China’s Heilongjiang Province

**DOI:** 10.1371/journal.pone.0102006

**Published:** 2014-07-07

**Authors:** Wei Zhao, Rongjun Wang, Weizhe Zhang, Aiqin Liu, Jianping Cao, Yujuan Shen, Fengkun Yang, Longxian Zhang

**Affiliations:** 1 Department of Parasitology, Harbin Medical University, Harbin, Heilongjiang, China; 2 College of Animal Science and Veterinary Medicine, Henan Agricultural University Zhengzhou, Henan, China; 3 National Institute of Parasitic Diseases, Chinese Center for Disease Control and Prevention, Key Laboratory of Parasite and Vector Biology, Ministry of Health, WHO Collaborating Centre for Malaria, Schistosomiasis and Filariasis, Shanghai, China; Cornell University, United States of America

## Abstract

Cattle are the main reservoir host of *C. andersoni*, which shows a predominance in yearlings and adults of cattle. To understand the subtypes of *C. andersoni* and the population genetic structure in Heilongjiang Province, fecal specimens were collected from 420 dairy cattle and 405 beef cattle at the age of 12–14 months in eight cattle farms in five areas within this province and were screened for the presence of *Cryptosporidium* oocysts by microscopy after Sheather’s sugar flotation technique. The average prevalence of *Cryptosporidium* spp. was 19.15% (158/825) and all the *Cryptosporidium* isolates were identified as *C. andersoni* by the SSU rRNA gene nested PCR-RFLP using *SspI*, *VspI* and *MboII* restriction enzymes. A total of 50 *C. andersoni* isolates were randomly selected and sequenced to confirm the RFLP results before they were subtyped by multilocus sequence typing (MLST) at the four microsatellite/minisatellite loci (MS1, MS2, MS3 and MS16). Four, one, two and one haplotypes were obtained at the four loci, respectively. The MLST subtype A4,A4,A4,A1 showed an absolute predominance and a wide distribution among the six MLST subtypes obtained in the investigated areas. Linkage disequilibrium analysis showed the presence of a clonal population genetic structure of *C. andersoni* in cattle, suggesting the absence of recombination among lineages. The finding of a clonal population genetic structure indicated that the prevalence of *C. andersoni* in cattle in Heilongjiang Province is not attributed to the introduction of cattle. Thus, prevention and control strategies should be focused on making stricter measures to avoid the occurrence of cross-transmission and re-infection between cattle individuals. These molecular data will also be helpful to explore the source attribution of infection/contamination of *C. andersoni* and to elucidate its transmission dynamics in Heilongjiang Province, even in China.

## Introduction

*Cryptosporidium* spp. are the important intestinal pathogens in both humans and animals with a global distribution. The parasites can cause diarrhea in both immune-compromised and immune-competent individuals, and show a high mortality in patients with HIV-infection [1]–[4]. Numerous studies of molecular characterizations of *Cryptosporidium* isolates confirm the presence of extensive genetic variations within the genus *Cryptosporidium*. To date, 26 species and more than 70 genotypes of *Cryptosporidium* have been identified with new genotypes being found, and 13 species and three genotypes of *Cryptosporidium* have been isolated from humans [5]–[8].

Various subtyping tools have been developed and proven to be useful in molecular epidemiological and population genetic studies for *Cryptosporidium* spp, with GP60 gene sequencing being the most commonly used tool for *Cryptosporidium* subtyping. However, gp60 subtyping is restricted to *C. parvum* and *C. hominis* as well as other *Cryptosporidium* species/genotypes, which are genetically closely related to both of them [5], [9]–[12]. A high-resolution multilocus sequence typing (MLST) technique has also been applied to characterize the genetics and population structure of *C. parvum* and *C. hominis* based on length polymorphism and single nucleotide polymorphism (SNP) [5], [13]–[15]. For *C. muris* and *C. andersoni* as rare *Cryptosporidium* species in humans, the genetic data were available only at a genotype level. Take *C. andersoni* for example, to date, the identification of human-derived *C. andersoni* isolates was only based on the analysis of SSU rRNA gene or COWP gene, including a recent study conducted in Shanghai, China, where all the 34 *Cryptosporidium*-positive patients were confirmed to be infected with *C. andersoni* [16]–[20]. Recently, an MLST tool used for subtyping *C. muri*s and *C. andersoni* has been established by Feng et al. [21]. It will be helpful in solving the problems on tracking the source of infection/contamination and elucidating transmission dynamics of human cryptosporidiosis caused by *C. muris* and *C. andersoni* by providing more genetic data.

Epidemiological data from different hosts have documented the host range or the host specificity of *C. andersoni*. Cattle are considered to be the main animal reservoir hosts of *C. andersoni*, although it is also occasionally detected in other animals such as bactrian camels, sheep, and hamsters [11], [22], [23]. Studies of age-associated distribution of *C. andersoni* in cattle reveal that *C. andersoni* is the predominant *Cryptosporidium* species responsible for cattle cryptosporidiosis in yearlings and adults [24]–[28]. Recent studies subtyped successfully *C. andersoni* isolates from cattle by MLST, including some isolates from a few areas in China [21], [29], [30]. In northeast’s China, Heilongjiang Province, cattle are one of the most important economic animals and have been reported to be infected with *C. andersoni*, even in preweaned calves [12], [31]. In addition, *C. andersoni* oocysts have been detected in raw wastewater from urban wastewater treatment plants [32]. The aims of the present study were to subtype C. *andersoni* isolates from yearlings of dairy cattle and beef cattle in Heilongjiang Province by MLST, and to elucidate population genetic structure of *C. andersoni* by diversity statistical test, and measurements of linkage disequilibrium. Meanwhile, we explored the relationship between MLST subtypes and breeds of cattle. The MLST data will be helpful to avoid or reduce the occurrence of cattle cryptosporidiosis in the investigated areas by making efficient control strategies based on characterization of population genetic structure. They will also be valuable to assess the risk that cattle infected with *C. andersoni* pose to humans by comparing population genetics of *C. andersoni* from humans and cattle in the future.

## Materials and Methods

### Ethics statement

This study was strictly performed in accordance with the recommendations of the Regulations for the Administration of Affairs Concerning Experimental Animals of Harbin Medical University/the Ministry of Health, China. All the fecal samples were obtained by the collection of feces excreted from cattle under the permission of farm owners to have their animals involved, with no specific permits being required by the authority. The protocol of the present study was reviewed and approved by the Animal Ethical Committee of Harbin Medical University (HMUIRB20130009).

### Collection of *C. andersoni* isolates

Between May 2012 and July 2013, a total of 825 fecal specimens (420 from dairy cattle and 405 from beef cattle) were randomly collected from yearlings in eight cattle farms in five areas within Heilongjiang Province, including Harbin, Qiqihar, Mudanjiang, Jiamusi and Daqing. Their ages ranged from 12 to 14 months. All the fecal samples were taken immediately from fresh feces deposited on the ground after cattle defecation, and Sheather’s sugar flotation technique was used to concentrate *Cryptosporidium* oocysts. The concentrates were detected by bright-field microscopy under ×400 and ×1,000. All the fecal samples microscopy-positive for *Cryptosporidium* oocysts were stored in 2.5% potassium dichromate at 4°C prior to being used in molecular biologic characterizations.

### DNA Extraction and molecular identification of *C. andersoni*

Potassium dichromate was washed off *Cryptosporidium* oocyst-positive fecal samples with distilled water by centrifugation at 1500 g for 10 minutes at room temperature four times. Genomic DNA was extracted from 200 mg of each fecal sample using a QIAamp DNA Mini Stool Kit (Qiagen, Hilden, Germany) according to the manufacturer recommended procedures. Eluted DNA was stored at −20°C until further use in PCR analysis. All DNA preparations were confirmed molecularly for the presence of *Cryptosporidium* spp. by a nested PCR amplification of an approximate 830 bp fragment of the SSU rRNA gene as previously described, followed by RFLP analysis with *Ssp*I, *Vsp*I and *Mbo*II restriction enzymes to determine *Cryptosporidium* species/genotypes [33]–[35]. Not all positive specimens were sequenced to further confirm the RFLP results, and we only sequenced *C. andersoni* isolates used for the MLST study.

### Molecular characterizations of *C. andersoni* at MS1, MS2, MS3 and MS16 loci

Approximate 30% of *C. andersoni* positive-samples of each farm were subtyped by amplifying the four minisatellite/microsatellite markers by nested PCRs, respectively. Target genes were MS1 coding for hypothetical protein, MS2 coding for 90 kDa heat shock protein, MS3 coding for hypothetical protein, and MS16 coding for leucine rich repeat family protein. The expected fragment lengths were approximate 550 bp, 450 bp, 530 bp and 590 bp, respectively, and primers and amplification conditions in nested PCR analysis were used as Feng et al described previously[21].

### Nucleotide sequence analysis

All purified secondary PCR products were directly sequenced with secondary PCR primers on an ABI PRISMTM 3730 DNA Analyzer (Applied Biosystems, USA), using a BigDye Terminator v3.1 Cycle Sequencing kit (Applied Biosystems, USA). Accuracy of the sequencing data was confirmed by sequencing in both directions and additional PCR products if necessary. All the gene sequences obtained in the present study were aligned with each other and reference sequences obtained from GenBank by the Basic Local Alignment Search Tool (BLAST) and Clustal X 1.83. *C. andersoni* subtypes were named according to the numbers in microsatellite/minisatellite repeats or/and nucleotide diversity in non-repeat regions at each locus (Table 1), and a novel subtype was designated if no identical sequences were obtained.

**Table 1:** Genetic characterizations of haplotypes of *C. andersoni* at MS1, MS2, MS3 and MS16 loci.

Haplotype	MS1			MS2				MS3					MS16	
	Accession No.^a^	Repeats^b^		Accession No.^a^	Repeat^b^	Polymorphic site		Accession No.^a^	Repeats^b^		Polymorphic site		Accession No.^a^	Repeat^b^
		a (n)	b (n)		c (n)	278	489		d (n)	e (n)	220	473		f (n)
A1	HM565067	3	14	HM565078	11	T	G	HM565095	2	2	A	C	HM565086	14
A2	JF732838	3	13	HM565079	10	T	G	HM565097	2	3	G	C	JF732872	12
A3	JF732836	3	12	HM565080	10	C	A	HM565094	2	3	G	T		
A4	JF732843	3	15	JF732853	10	C	G	HM565096	2	2	G	C		
A5	JF732845	3	16	JF732851	11	C	G							
A6	JF732846	3	17											

a Accession numbers of all the reference sequences from GenBank.

b The small letters a to f in the horizontal subheading represent the repeats at the four loci: a = (TAAAGGGCGAGA); b = (GAACGAGATAGG); c = (CCATACCTC); d = (TGTTGGTGTTGCTGT); e = (TGCTGCAGCTGC); f = (CTTCTTCAT).

### Data analysis

DnaSP version 5.10.01 (http://www.ub.edu/dnasp/) was used to analyze the genetic diversity of *C. andersoni* isolates. Sequences from four loci were combined in a single contig and analyzed for linkage disequilibrium (LD) across the entire composite sequence by DnaSP 5.10.01 [36]. D’ was calculated for all pairs of sites. Both the two-tailed Fisher’s exact test and the *χ* ^2^ test were used to determine significance of the associations between polymorphic sites. The average LD was estimated by using the Z*n*S statistic, which averages LD over all pairwise comparisons for S polymorphisms in N sequences.

## Results

### Prevalence of *C. andersoni* in yearlings

A total of 420 and 405 fecal samples of 12–14-month-old dairy cattle and beef cattle, respectively, were screened for the presence of *Cryptosporidium* oocysts by microscopy. The average prevalence of *Cryptosporidium* spp. was 19.15% (158/825). *Cryptosporidium* oocysts were detected in 20.71% (87/420) dairy cattle and 17.53% (71/405) beef cattle, with no statistical difference in the prevalence between them (*χ* ^2^ = 1.35, *P* = 0.25). Meanwhile, cattle were found to be infected with *Cryptosporidium* in all eight farms from the five investigated areas (Table 2). All the *Cryptosporidium* isolates were molecularly confirmed as *C. andersoni* by SSU rRNA nested PCR-RFLP using *SspI*, *VspI* and *MboII* restriction enzymes. Additionally, 50 *C. andersoni* isolates randomly selected for the MLST analysis were sequenced to further confirm the RFLP results.

**Table 2:** Prevalence and MLST subtypes of *C. andersoni* from dairy cattle and beef cattle.

Collection site	Dairy cattle			Beef cattle		
	Positive no./Examined no.(%)	Subtyped no./Amplified no.^a^	MLST subtypes (n)^b^	Positive no./Examined no. (%)	Subtyped no./Amplified no.^a^	MLST subtypes (n)^b^
Daqing	22/112 (19.64)	6/7	A4,A4,A4,A1 (5), A2,A4,A4,A1 (1)	11/89 (12.36)	3/3	A5,A4,A4,A1 (2), A1,A4,A4,A1 (1)
Harbin	13/83 (15.66)	4/4	A4,A4,A4,A1 (4)	NE		
Jiamusi	NE			7/108 (6.48)	2/2	A4,A4,A4,A1 (2)
Mudanjiang	40/153 (26.14)	10/12	A4,A4,A4,A1 (6), A2,A4,A4,A1 (1), A2,A4,A2,A1 (1), A4,A4,A2,A1 (2)	13/142 (9.15)	3/4	A4,A4,A4,A1 (2), A1,A4,A4,A1 (1)
Qiqihaer	12/72 (16.67)	4/4	A4,A4,A4,A1 (4)	40/66 (60.60)	13/14	A4,A4,A4,A1 (10), A5,A4,A4,A1 (3)
Total	87/420 (20.71)	24/27	A4,A4,A4,A1 (19), A2,A4,A4,A1 (2), A2,A4,A2,A1 (1), A4,A4,A2,A1 (2)	71/405 (17.53)	21/23	A4,A4,A4,A1 (14), A5,A4,A4,A1 (5), A1,A4,A4,A1 (2)

Note: NE = not examined.

a Subtyped no. indicating the number of *C. andersoni* isolates subtyped successfully by PCR at all the four loci (MS1, MS2, MS3 and MS16); Amplified no. indicating the number of *C. andersoni* isolates analyzed by PCR at all the four loci (MS1, MS2, MS3 and MS16).

b The haplotypes were arranged in accordance with the order of gene loci amplified, MS1, MS2, MS3 and MS16.

### Multilocus subtypes and polymorphism of *C. andersoni*

Approximate 30% of *C. andersoni* isolates were collected from each cattle farm and a total of 50 *C. andersoni* isolates (27 from dairy cattle and 23 from beef cattle) were used for analysis of MLST subtypes. At the four microsatellite/minisatellite loci (MS1, MS2, MS3 and MS16), 47, 48, 46 and 45 DNA preparations were sequenced successfully, respectively, and four, one, two and one haplotypes were identified, respectively. Representative nucleotide sequences were deposited in the GenBank under accession numbers KJ001678 to KJ001689.

A total of 45 out of 50 *C. andersoni* isolates were successfully subtyped at all the four loci, with six MLST subtypes being found. The MLST subtype A4,A4,A4,A1 showed a predominance, accounting for a high prevalence (73.33%, 33/45) in cattle with 79.16% (19/24) for dairy cattle versus 66.67% (14/21) for beef cattle. This subtype appeared in seven out of eight cattle farms, showing an extensive distribution in the investigated areas. However, the other three MLST subtypes, A2,A4,A4,A1 (n = 2), A2,A4,A2,A1 (n = 1) and A4,A4,A2,A1 (n = 2) were only found in dairy cattle while the remaining two MLST subtypes, A5,A4,A4,A1 (n = 5) and A1,A4,A4,A1 (n = 2) were only found in beef cattle (Table 2).

Sequence data of all four loci were concatenated making a multilocus gene of 1864 bp length. Genetic diversity of sequences was analyzed using DnaSP 5.10.01. The 45 sequences produced 18 polymorphic sites and two haplotypes with one haplotype diversity of 0.202±0.073, nucleotide diversity of 0.00200, and average number of nucleotide differences of 3.636.

### Linkage disequilibrium analysis

A total of 45 *C. andersoni* isolates subtyped successfully at all the four loci were included in LD analysis. To examine LD within and between loci, we analyzed pairwise associations of polymorphism across the concatenated sequences. LD was estimated using the *Zn*S statistic, which calculates the average correlation among alleles in all pairs of polymorphic sites. *Zn*S value of 1.0000 indicated that the *C. andersoni* isolates had a clonal population structure, suggesting the absence of recombination among lineages, which was supported by the D’ statistic (Figure S1).

## Discussion

Cattle are considered as the main reservoir hosts of *C. andersoni* although the parasite has also been isolated from sheep, bactrian camels and hamsters [11], [22], [23]. Using the MLST tool for subtyping *C. muris* and *C. andersoni* developed by Feng et al. (2011) [21], a total of 14 MLST subtypes have been identified in *C. muris* isolates from animals and humans [21], [29], while 18 MLST subtypes have been found in *C. andersoni* isolates from animals with 14 subtypes in cattle [21], [29], [30] (details seen in Table 3). In the present study, we investigated the occurrence of *Cryptosporidium* spp. in 12–14-month-old yearlings of dairy cattle and beef cattle by microscopy. The average prevalence of *Cryptopsoridium* spp. was 19.15% (157/825) with 20.71% (87/420) for dairy cattle versus 17.35% (71/405) for beef cattle, and all the *Cryptosporidium* isolates were identified as *C. andersoni.* Among the 50 *C. andersoni* isolates selected for MLST analysis, only 45 *C. andersoni* isolates were successfully subtyped at all the four microsatellite/minisatellite loci (MS1, MS2, MS3 and MS16) and six MLST subtypes have been obtained (Table 2). The MLST subtype A4,A4,A4,A1 was the most prevalent and the most widespread compared to other five subtypes in the investigated areas, based on the fact that this subtype could be found in 73.33% (33/45) of *C. andersoni* isolates and in 87.5% (7/8) of cattle farms. According to the present and previous data, *C. andersoni* MLST subtype (A4,A4,A4,A1) was also the most common in cattle in China among 10 subtypes identified (Table 4). Meanwhile, *C. andersoni* MLST subtypes in China were noticed to be different from those found in cattle in other countries, accounting for subtypes A2,A3,A2,A1, A2,A3,A1,A1 and A2,A3,A4,A1 in the USA, A2,A3,A4,A1 in Canada, and A2,A3,A4,A1 and A1,A3,A4,A1 in the Czech Republic [21]. The differences in genetic characterizations of *C. andersoni* might be related to the geographic separation just like *C. parvum* and *C. hominis* [5]. The results above suggested that occurrence of *C. andersoni* in cattle in China is not attributable to the introduction of cattle.

**Table 3:** MLST subtypes of *C. andersoni* from different hosts worldwide.

Host	Subtyped no.	MS1	MS2	MS3	MS16	Reference
sheep	1	A2	A4	A2	A1	[29]
	1	A2	A5	A2	A1	[29]
hamster	2	A3	A4	A2	A2	[29]
bactrian camel	1	A6	A4	A2	A1	[29]
	1	A6	A5	A2	A1	[29]
cattle	3	A1	A2	A4	A1	[21]
	1	A1	A3	A4	A1	[21]
	34	A1	A4	A4	A1	[29], [30]; this study
	2	A2	A1	A2	A1	[21]
	1	A2	A1	A3	A1	[21]
	1	A2	A3	A1	A1	[21]
	1	A2	A3	A2	A1	[21]
	3	A2	A3	A4	A1	[21]
	6	A2	A4	A2	A1	[29], [30]; this study
	6	A2	A4	A4	A1	[29], [30]; this study
	1	A3	A4	A4	A1	[29]
	3	A4	A4	A2	A1	[30]; this study
	82	A4	A4	A4	A1	[29], [30]; this study
	6	A5	A4	A4	A1	[29]; this study

**Table 4:** MLST subtypes of *C.andersoni* in cattle in different provinces of China.

Location	Subtyped no.	MLST subtype (no.)	Reference
Guangxi	4	A1,A4,A4,A1 (2); A2,A4,A2,A1 (1); A4,A4,A4,A1 (1)	[29]
Heilongjiang	50	A4,A4,A4,A1 (37); A5,A4,A4,A1 (5); A2,A4,A4,A1 (2); A2,A4,A2,A1 (2)A4,A4,A2,A1 (2); A1,A4,A4,A1 (2)	[29]; this study
Henan	22	A4,A4,A4,A1 (9); A1,A4,A4,A1 (3); A1,A2,A4,A1 (3);A2,A4,A2,A1 (2); A2,A1,A2,A1 (2); A2,A1,A3,A1 (1);A3,A4,A4,A1 (1); A5,A4,A4,A1 (1)	[29], [21]
Jilin	1	A4,A4,A4,A1 (1)	[29]
Shaanxi	57	A4,A4,A4,A1 (26); A1,A4,A4,A1 (26); A2,A4,A4,A1 (3);A2,A4,A2,A1 (1); A4,A4,A2,A1 (1)	[30]
Shanxi	5	A4,A4,A4,A1 (5)	[29]
Sichuan	5	A4,A4,A4,A1 (3); A2,A4,A4,A1 (1); A1,A4,A4,A1 (1)	[29]
Total	144	A4,A4,A4,A1 (82); A1,A4,A4,A1 (34); A2,A4,A4,A1 (6); A2,A4,A2,A1 (6); A5,A4,A4,A1 (6); A4,A4,A2,A1 (3);A3,A4,A4,A1 (1); A1,A2,A4,A1 (3); A2,A1,A2,A1 (2);A2,A1,A3,A1 (1)	

All but MLST subtype A4,A4,A4,A1 were subtypes apparently associated to either dairy or beef cattle (Table 2). However, three subtypes (A2,A4,A4,A1, A2,A4,A2,A1 and A4,A4,A2,A1) in dairy cattle and one subtype (A1,A4,A4,A1) in beef cattle obtained in the present study were also described in beef cattle and dairy cattle in Shaanxi, China, respectively [30], suggesting that cross transmission of *C. andersoni* might occur between two breeds of cattle. However, to date, the MLST subtype A5,A4,A4,A1 has only been found in beef cattle in Heilongjiang and Henan of China [29]. Whether MLST subtypes are related to cattle breeds needs to be confirmed by a larger sample size of *C. andersoni* from cattle. The fact that the MLST subtype A2,A4,A2,A1 of *C. andersoni* in cattle was also reported in sheep further evidences that *C. andersoni* might circulate between cattle and sheep [29].

The intra-genetic variations of cattle-derived *C. andersoni* were observed in the present study based on multilocus DNA sequence analysis of a 1864 bp length fragment for 45 *C. andersoni* isolates by DnaSP. We could see 18 polymorphic sites and two haplotypes, with one haplotype diversity of 0.202±0.073, nucleotide diversity of 0.00200, and average number of nucleotide differences of 3.636. In fact, genetic polymorphism has been described within *C. andersoni* at a subtype level. The present data, together with previous studies of MLST subtypes of *C. andersoni*, revealed the nucleotide differences of each haplotype at any locus in the number of microsatellite/minisatellite repeats or/and in the base variation in the non-repeat regions. In general, nucleotide sequences are the most conservative at the MS16 locus compared to the other three loci analyzed based on the fact that only two haplotypes have been currently found at this locus: A1 in cattle, sheep and camels, and A2 only in hamsters (Table 3). Thus, we can subtype *C. andersoni* isolates from a specific host at the other loci except MS16 locus to study intra-genetic variations of this parasite. Currently, six, five and four haplotypes of *C. andersoni* from different hosts have been identified worldwide at MS1, MS2 and MS3 loci, respectively (Table 3). At the MS1 locus, the genetic difference of six subtypes of *C. andersoni* (A1 to A6) is totally reflected in the copy number of microsatellite/minisatellite repeats. In contrast, at the MS2 and MS3 loci, the haplotypes of *C. andersoni* are named based on the copy number of microsatellite/minisatellite repeats, and in the case of the same number of repeats, the haplotypes of *C. andersoni* are distinguished from each other according to base variations in the non-repeat regions. The genetic characterizations of each haplotype at each locus has been summarized in Table 1.

In the present study, significant LD (*Zn*S value of 1.0000) showed that *C. andersoni* had a clonal population genetic structure. The result suggested the absence of recombination among lineages of *C. andersoni* in cattle in Heilongjiang Province, which was consistent with *C. andersoni* isolates from other areas in China [29], [30]. A clonal population genetic structure indicates the multilocus subtypes are relatively stable in time and place, and thus can be used effectively in the longitudinal tracking of the transmission and in the investigation of cryptosporidiosis outbreaks caused by *C. andersoni* in the areas investigated. Wang et al. (2012) conducted LD analysis of *C. andersoni* population in cattle in China [29]. When the isolates showing the same MLST subtype were scored as one individual, the result of *I^S^_A_* value (*I^S^_A_* = 0.0290, *V_D_*<*L*) suggested that cattle-derived *C. andersoni* isolates had an epidemic population structure, which might result from the clonal expansion of two MLST subtypes (A4,A4,A4,A1 and A1,A4,A4,A1) [29].

In conclusion, six MLST subtypes of *C. andersoni* were found in cattle in Heilongjiang Province, with the subtype A4,A4,A4,A1 being predominant in both dairy and beef cattle. The finding of the same MLST subtypes in dairy cattle, beef cattle and sheep in the present and previous studies suggest the likelihood of cross transmission of *C. andersoni* between these hosts [29], [30]. The MLST subtype A5,A4,A4,A1 has only been found in beef cattle. Nevertheless, we cannot draw a definitive conclusion about the relationship between *C. andersoni* MLST subtypes and breed of cattle due to the small sample size. The characterization of a clonal population genetic structure of *C. andersoni* indicates that the prevalence of *C. andersoni* in cattle is not attributed to the introduction of cattle. Thus, a key component to avoid or reduce cattle cryptosporidiosis is to prevent the occurrence of cross-transmission and re-infection between cattle individuals by making effective control strategies. The MLST data will be helpful to explore the source attribution of infection/contamination of human cryptosporidiosis caused by *C. andersoni* and its transmission dynamics in Heilongjiang Province, even in China.

## Supporting Information

Figure S1 **Linkage disequilibrium among different populations by DnaSP analysis of concatenated sequences from four genetic loci.** Sequences from four loci were combined in a single contig and analyzed for linkage disequilibrium (LD) across the entire composite sequence by DnaSP 5.10.01 software.
(TIF)
