# Supplementary figures and images for: MLST Subtypes and Population Genetic Structure of Cryptosporidium andersoni from Dairy Cattle and Beef Cattle in Northeastern China’s Heilongjiang Province
Source: PLoS One. 2014 Jul 7;9(7):e102006. doi: 10.1371/journal.pone.0102006 (PMC4084942; doi:10.1371/journal.pone.0102006)

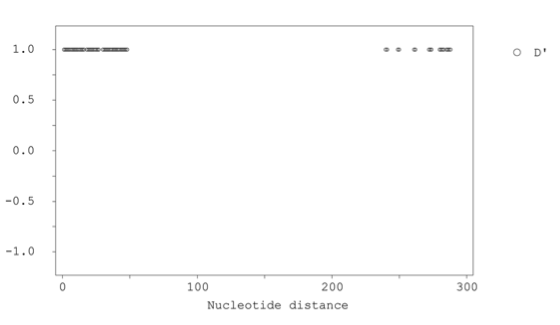

Supplement: Figure S1 — Linkage disequilibrium among different populations by DnaSP analysis of concatenated sequences from four genetic loci. Sequences from four loci were combined in a single contig and analyzed for linkage disequilibrium (LD) across the entire composite sequence by DnaSP 5.10.01 software. (TIF) [file pone.0102006.s001.tif]
